# Hysteroscopy in the new media: quality and reliability analysis of hysteroscopy procedures on YouTube™

**DOI:** 10.1007/s00404-023-07172-9

**Published:** 2023-08-12

**Authors:** Alessandro Libretti, Salvatore Giovanni Vitale, Stefania Saponara, Christian Corsini, Carmen Imma Aquino, Federica Savasta, Eleonora Tizzoni, Libera Troìa, Daniela Surico, Stefano Angioni, Valentino Remorgida

**Affiliations:** 1 Department of Gynaecology and Obstetrics, University Hospital Maggiore Della Carità, Novara, Italy; 2 School of Gynaecology and Obstetrics, University of Eastern Piedmont, Novara, Italy; 3 Division of Gynecology and Obstetrics, Department of Surgical Sciences, University of Cagliari, Cagliari, Italy; 4 Division of Experimental Oncology/Unit of Urology, URI, IRCCS San Raffaele Hospital, Milan, Italy; 5 Vita-Salute San Raffaele University, Milan, Italy; 6 Department of Gynaecology and Obstetrics, Filippo Del Ponte Hospital, Varese, Italy

**Keywords:** Hysteroscopy, YouTube, mDISCERN, GQS, Web information

## Abstract

**Background:**

Hysteroscopy plays a crucial role in diagnosing and managing various intrauterine pathologies. However, its execution can be influenced by patients’ perception and understanding, which are often shaped by digital resources such as YouTube^TM^. Given its popularity and accessibility, YouTube^TM^ has the potential to greatly influence patients’ knowledge and expectations about this procedure, highlighting the need for accurate and reliable information.

**Purpose:**

This study aims to assess the reliability and quality of hysteroscopy information available to patients on YouTube^TM^. Understanding the nature of information patients’ access can help address their fears and potential misunderstandings about the procedure, consequently reducing the likelihood of suspension or postponement due to anxiety.

**Methods:**

A comprehensive analysis of YouTube^TM^ was conducted, simulating the search process of a patient seeking information about hysteroscopy. The study evaluated the reliability and quality of 90 out of the first 100 hysteroscopy-related videos on YouTube^TM^, scored by four gynecologists—two experienced hysteroscopists and two trainees. The videos were assessed for reliability and quality using the mDISCERN and Global Quality Scale (GQS) scores.

**Results:**

The average mDISCERN and GQS scores for the evaluated videos were below the optimal three points, highlighting the lack of fluency, comprehensiveness, and reliability of the available information. Notably, while videos produced by experts, including doctors and professional channels, had higher scores, they still fell short of the minimum score of 3. These videos also were not considered more suitable for either patients or trainees. Videos that were assessed as reliable (mDISCERN ≥ 3) were observed to be longer and were more frequently produced by doctors. These videos were suggested more to trainees rather than patients. Similarly, videos deemed as fluent and comprehensive (GQS ≥ 3) were longer and were more often recommended to patients.

**Conclusions:**

While YouTube^TM^ is a widely used source of medical information, the quality and reliability of hysteroscopy videos on the platform are poor. The strategic use of selected, high-quality hysteroscopy videos can enhance procedure success and alleviate patient fears. However, the unsupervised discovery of information by patients could potentially lead to procedure failure or an elevated level of stress due to misleading or incorrect information.

## What does this study add to the clinical work?

**Table:** 

This study underscores the crucial role of scrutinizing digital sources of patient education, especially popular platforms like YouTube, for the reliability and quality of medical information. It highlights the need for healthcare providers to proactively recommend reliable, comprehensible resources to patients undergoing hysteroscopy, thus minimizing misinformation-induced stress and procedure failure.	

## Introduction

The advent and widespread use of the internet have facilitated easy access to a vast array of online resources, transforming them into a significant wellspring of health information for patients and caregivers [1–7]. Evidence from the Health Information National Trends Survey (HINTS) underscores this trend, highlighting an exponential increase in individuals seeking health-related information online [8]. Pew Research Center surveys corroborate this, reporting that three-quarters of online health information seekers had treatment choices shaped by the insights gleaned from their online findings [3]. Similarly, in 2020, 55% of EU citizens aged 16–74 reported that they had sought online health information relating to injury, disease, nutrition, health improvement, or similar topics [9].

YouTube™ has emerged as a predominant platform among the myriad of online resources. In 2022, YouTube™ counted over 2.56 billion users accessing its video content worldwide [10]. However, YouTube™’s model does not regulate the credibility of content creators, potentially leading to the dissemination of unverified or non-expert content. This is further exacerbated by the lack of a peer-review process for content uploaded on YouTube™, allowing registered users to post content at their discretion.

In light of this scenario, concerns have been raised by healthcare providers and regulatory bodies regarding the accuracy and quality of the accessible information, particularly given the prevalent sharing of anecdotal experiences and personal viewpoints [11]. This becomes particularly significant when considering specific medical procedures, such as hysteroscopy, susceptible to misinformation and misunderstanding.

Hysteroscopy, an endoscopic surgical procedure, offers direct visualization of the uterine cavity and allows biopsy of suspected lesions [12]. It is the gold standard procedure for evaluating and managing intrauterine pathologies, abnormal uterine bleeding, infertility, intrauterine retained products of conception, suspected Müllerian anomalies and Caesarean scar defects (isthmocele) [12–21].

Initially performed only in the operating room under general anesthesia, hysteroscopy has gradually transitioned to an office setting due to advancements in technology, such as the miniaturization of endoscopes and improvements in optics and surgical techniques [22, 23]. This transition has helped mitigate the need for hospital admission, preoperative tests, and general or regional anesthesia, reducing the postsurgical recovery period, overall procedure cost, and complication rate [24]. However, managing patient anxiety remains a critical challenge in completing office hysteroscopy, given that it can intensify pain perception and limit procedural tolerance [25].

Despite the widespread application of hysteroscopy and the common use of YouTube™ as a health information resource, no published studies have analyzed the information available on YouTube™ regarding this procedure.

Therefore, our study aims to examine the reliability and quality of hysteroscopy-related information on YouTube™. We analyzed YouTube™ content, simulating the information-seeking process of a patient preparing to undergo a hysteroscopy. The goal is to understand how patients gather information about the procedure and how to alleviate their fears before undergoing hysteroscopy to avoid the necessity of procedure suspension or postponement. Our analysis can shed light on the influence of YouTube™ as a medical information dissemination platform and contribute valuable insights to enhance patient preparedness for hysteroscopy.

## Materials and methods

### Videos selection

The YouTube™ search was conducted on June 3, 2023, using the term “hysteroscopy”.

**Fig. 1:**
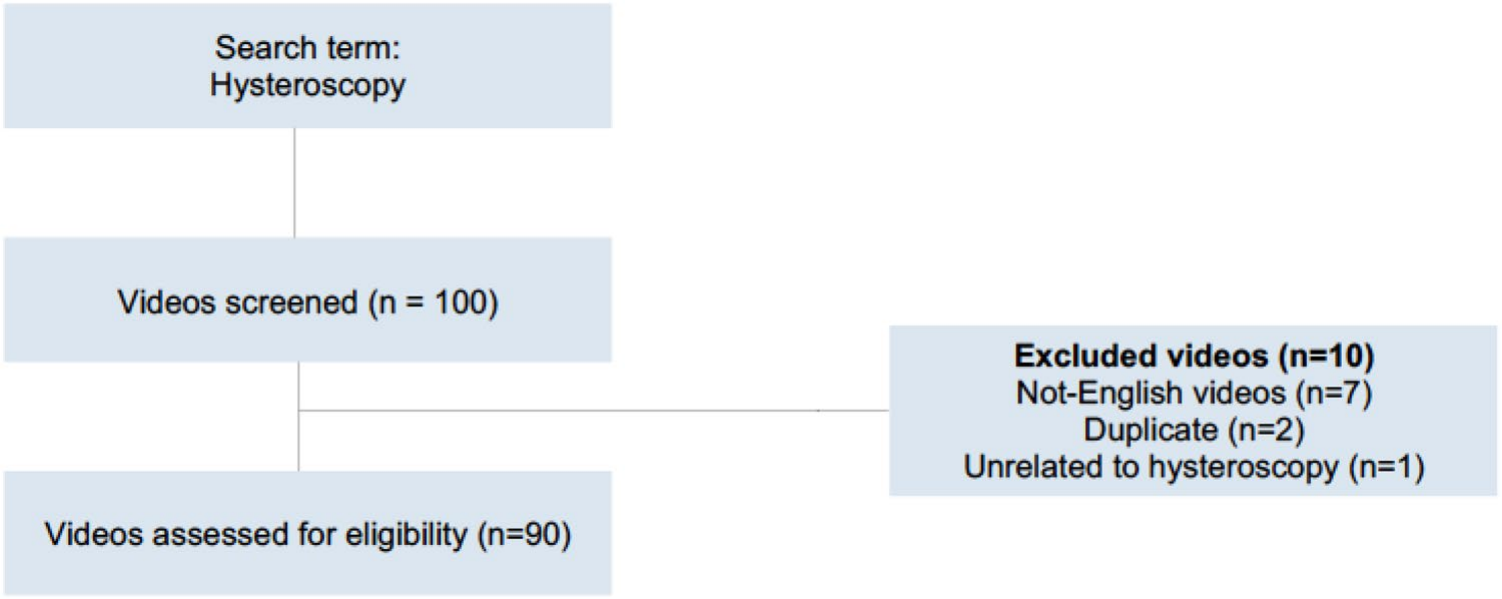
Methodology of selection of YouTube™ videos

The YouTube™ setting was “global”, and no filters were used to faithfully reproduce the search of a hypothetical patient proposed with the hysteroscopy procedure. We limited our search to the first 100 videos in line with existing literature, suggesting that only 8% of internet users continue their search after bending this number [26]. Our selection process was based on several inclusion and exclusion criteria. To qualify for inclusion, videos had to be in English or without audio and had to primarily concern hysteroscopy. Conversely, exclusion criteria encompassed videos in languages other than English, videos unrelated to hysteroscopy, and duplicates. Of the 100 videos evaluated, 90 met the inclusion criteria and were selected for further analysis. The remaining ten videos were eliminated from our review for the following reasons: 7 videos were in a language other than English, 2 were duplicates, and 1 video was unrelated to hysteroscopy (Fig. 1). The complete playlist of the 90 selected videos is available at the following link: https://www.youtube.com/playlist?list=PLPGE3LF2gQpk-9U2qYbN259_aLqgl96Qv.

Ethics committee approval was not required as this study included no human participants, and the videos were publicly accessible.

### Data collection

A data extraction process was conducted on the selected 90 videos to gather a wide array of information. The specific parameters extracted from each video included:

- Language: the language in which the video was presented was documented. In alignment with our inclusion criteria, all the selected videos were in English.
- Production source: videos were classified based on their production source, including professional entities like doctors, professional channels, hospitals and non-professional sources such as internet or television platforms or those created by patients without professional backing.
- Purpose of the video: each video was categorized as either ‘informative’ or ‘sponsor’. The latter category pertained to videos with an apparent commercial purpose.
- Presenter’s gender: the gender of the video presenter was recorded.
- Time on YouTube™: the period for which each video has been accessible on YouTube™ was determined, measured in months.
- Video duration: the length of each video was documented, measured in seconds.
- Viewer engagement: several metrics were evaluated to assess viewer engagement, including the total number of views, number of likes, number of comments, and the number of subscribers of the uploader.

### Evaluation of video reliability and content quality

The quality and reliability of the videos were evaluated by a team composed of two experienced gynecologists (SGV and VR), who have each performed more than 100 diagnostic and operative hysteroscopic procedures, and two trainees in Obstetrics and Gynecology (AL and SS). To ensure the scientific reliability of the videos, we used the modified DISCERN scale [27]. Initially, this scale was designed by Charnock et al.to evaluate written health information [28]. The modified version (mDISCERN) consists of a five-question questionnaire (Table 1), with each affirmative response garnering one point towards a maximum of five [27]. Videos achieving an mDISCERN total score of three or above were deemed to provide reliable health information.

**Table 1:** Modified DISCERN tool used to evaluate the reliability of videos (1 point is given for every Yes and 0 points for No) [27]

Item	Questions
1	Are the aims clear and achieved?
2	Are reliable sources of information used? (i.e., publication cited, speaker is specialist)
3	Is the information presented balanced and unbiased?
4	Are additional sources of information listed for patient reference?
5	Are areas of uncertainty mentioned?

In addition, we used the Global Quality Scale (GQS) to grade the overall quality of the videos [29]. The GQS, a 5-point scale (Table 2), was developed to evaluate the fluency and comprehensiveness of information on the web [29]. A higher GQS score denotes content of better quality and informative value. Any video that scored three or more on the GQS was deemed to offer higher quality health information. The four operators independently but concurrently viewed and scored the videos during a five-days section.

**Table 2:** Global Quality Scale (GQS) criteria used to score videos quality [29]

Items	Characteristics
1	Low quality, low flow, most information missing, not beneficial for patients
2	Generally low quality and low flow of information, some listed information and many important issues are missing, very limited use for patients
3	Moderate quality, sub optimal flow of information, some important information are sufficiently discussed but some are poorly discussed: only somewhat useful for patient
4	Good quality and generally good information flow. Most of the relevant information is listed, but some topics are not covered, useful for patients
5	Excellent quality and information flow, very useful for patients

The team independently and simultaneously watched and scored the videos over five days. Each team member also responded to two final questions: “Would you recommend this video to patients?” and “Would you recommend this video to resident doctors?”

### Statistical analysis

The normal distribution of data was tested with the Shapiro–Wilk test. Data are presented as medians (interquartile range; IQR) for continuous variables and frequencies (proportions) for categorical variables. Data from general features and results of the assessment of the videos are presented as mean, standard deviation (SD), minimum (min) and maximum (max) for each variable. Data are presented stratified according to the production source (experts vs. others), mean mDISCERN, mean GQS and aim of the video (informative vs. sponsor). General features of the videos, reliability of the videos content and global quality were compared among the groups with the Wilcoxon–Mann–Whitney test for continuous variables and the Chi-square test for categorical variables, respectively. Statistical analyses were performed using R studio Inc. (Boston, MA, USA) integrated development environment for R software v. 3.5.3 (2016). All tests were two-sided, and the statistical significance level was determined at *p* < 0.05.

## Results

The general characteristics of the 90 videos selected for the study, including their upload duration, length, views, likes, and the number of subscribers to the uploader, as well as the evaluation results (mDISCERN and GQS scores), are provided in Table 3. The mean score for the two scales (mDISCERN and GQS) did not satisfy the minimum of 3 points, attesting to the low fluency, comprehensiveness (GQS) and reliability (mDISCERN) of information presented in the videos for both residents and specialists. Nevertheless, specialists provided higher average scores than trainees on both scales. Specifically, the overall mean mDISCERN score was 2.43 ± 0.79, with means of 2.38 ± 1.01 for residents and 2.47 ± 0.77 for specialists. The mean minimum and maximum scores ranged from 0.25 to 4.00 overall, 0.50 to 4.50 for residents and 0 to 4.00 for specialists.

**Table 3:** General features and assessment results of the analyzed videos

Video features	Mean ± SD (min, max)
Duration of upload on YouTube (days)	1923 ± 1527 (133, 6473)
Video length (s)	864 ± 1820 (16, 13,301)
Number of views (*n*)	95,187 ± 259,073 (36, 1,876,542)
Number of likes (*n*)	377 ± 896.40 (0, 6288)
Number of subscribers of the uploader (*n*)	209,287 ± 719,351 (4, 6,002,000)
mDISCERN score	2.43 ± 0.79 (0.25, 4.00)
- Residents	2.38 ± 1.01 (0.50, 4.50)
- Specialists	2.47 ± 0.77 (0, 4.00)
GQS score	2.64 ± 0.83 (1, 4.75)
- Residents	2.53 ± 0.93 (1, 5.00)
- Specialists	2.74 ± 0.90 (1, 4.50)

*mDISCERN* modified DISCERN, *GQS* Global Quality Score, *SD* standard deviation

In terms of GQS scores, the overall mean was 2.64 ± 0.83. Residents gave a mean score of 2.53 ± 0.93, and specialists gave a mean of 2.74 ± 0.90. The mean minimum and maximum scores ranged from 1 to 4.75 overall, 1 to 5.00 for residents, and 1 to 4.50 for specialists.

We divided the video sources into two categories: Experts, which included Doctors (*n* = 39), Professional Channels (*n* = 26), Hospitals (*n* = 18), and Patients (*n* = 7). The features and results of the assessment were compared across these categories, as presented in Table 4.

**Table 4:** Comparison of video features and assessment scores between videos produced by experts and patients

Features/outcome	Experts (Doctors = 39, professional channels = 26, hospitals = 18)	Patients (*n* = 7)	*P*-value
Duration of upload on YouTube (days)			
Median [Q1, Q3]	1460 [820, 2370]	1920 [1430, 1940]	0.65
Video length (sec)			
Median [Q1, Q3]	286 [124, 616]	684 [657, 930]	0.71
Nr. views			
Median [Q1, Q3]	14,800 [1580, 38,000]	59,400 [16,200, 203,000]	0.72
Nr. comments			
Median [Q1, Q3]	7.00 [1.00, 25.0]	20.0 [2.75, 152]	0.29
Nr. like			
Median [Q1, Q3]	77.0 [8.25, 248]	424 [60.0, 2190]	0.19
Nr. Subscribers			
Median [Q1, Q3]	11,500 [832, 70,800]	15,300 [10,700, 297,000]	0.76
mDISCERN total			
Median [Q1, Q3]	2.50 [2.25, 3.00]	1.00 [0.750, 1.13]	** < 0.001**
< 3	54 (65%)	7 (100%)	0.14
≥ 3	29 (35%)	0 (0%)	
mDISCERN residents			
Median [Q1, Q3]	2.50 [2.00, 3.00]	0.50 [0.50, 1.00]	** < 0.001**
< 3	48 (58%)	7 (100%)	0.07
≥ 3	35 (42%)	0 (0%)	
mDISCERN specialists			
Median [Q1, Q3]	2.50 [2.50, 3.00]	1.00 [1.00, 1.25]	** < 0.001**
< 3	57 (69%)	7 (100%)	0.19
≥ 3	26 (31%)	0 (0%)	
GQS total			
Median [Q1, Q3]	2.75 [2.25, 3.13]	1.50 [1.38, 1.75]	** < 0.001**
< 3	52 (63%)	7 (100%)	0.11
≥ 3	31 (37%)	0 (0%)	
GQS residents			
Median [Q1, Q3]	2.50 [2.00, 3.00]	1.50 [1.50, 1.75]	**0.001**
< 3	56 (67%)	6 (86%)	0.56
≥ 3	27 (33%)	1 (14%)	
GQS specialists			
Median [Q1, Q3]	3.00 [2.50, 3.50]	1.50 [1.25, 1.50]	** < 0.001**
< 3	39 (50%)	7 (100%)	**0.02**
≥ 3	44 (50%)	0 (0%)	
Suggested to patients?			
No	74 (89%)	7 (100%)	0.79
Yes	9 (11%)	0 (0%)	
Suggested to young trainees/resident doctors?			
No	76(92%)	7 (100%)	0.95
Yes	7 (8%)	0 (0%)	

Bold values indicate statistically significant results (*p*-value < 0.05)

*mDISCERN* modified DISCERN, *GQS* Global Quality Score

For most metrics related to video characteristics (duration of upload, length, number of views, comments, likes, and subscribers), the median values were generally higher for the patient group than the expert group. However, the *p*-values associated with these comparisons were relatively high (ranging from 0.19 to 0.76), indicating no statistically significant differences between the two groups in these respects.

Videos produced by experts received a statistically significant higher score from both specialists and trainees in the two scales of reliability of information (mDISCERN) and fluency and comprehensiveness (GQS). Despite this, the average scores of GQS and mDISCERN remained below 3 points, even for expert-produced content, signifying low reliability and comprehensiveness. Regarding the suggestion of videos to patients and resident doctors, most videos in both groups (92% for experts and 89% for patients) were not suggested.

The comparison of the general features of the videos according to mDISCERN is presented in Table 5. The videos with a higher mDISCERN score (≥ 3) tended to have a significantly longer median duration (459 s compared to 259 s).

**Table 5:** Comparison of video features according to mDISCERN score

	mDISCERN < 3 (*N* = 61)	mDISCERN ≥ 3 (*N* = 29)	*P*-value
Duration of upload on YouTube (days)			
Median [Q1, Q3]	1550 [920, 3280]	1190 [776, 2190]	0.42
Video length (sec)			
Median [Q1, Q3]	259 [111, 583]	459 [292, 2050]	**0.03**
Nr. views			
Median [Q1, Q3]	17,300 [1850, 46,400]	10,700 [1300, 50,400]	0.17
Nr. comments			
Median [Q1, Q3]	6.00 [1.00, 22.0]	9.00 [1.00, 75.0]	0.12
Nr. likes			
Median [Q1, Q3]	83.0 [15.0, 252]	131 [7.00, 449]	0.81
Nr. Subscribers			
Median [Q1, Q3]	14,300 [570, 90,300]	9850 [1510, 44,300]	0.62
Production			**0.02**
Doctors	20 (33%)	19 (66%)	
Professional channels	21 (34%)	5 (17%)	
Hospitals	13 (21%)	5 (17%)	
Patients	7 (11%)	0 (0%)	
Suggested to patients?			0.23
No	57 (93%)	24 (83%)	
Yes	4 (7%)	5 (17%)	
Suggested to young trainees/resident doctors?			**0.006**
No	60 (98%)	23 (79%)	
Yes	1 (2%)	6 (21%)	

Bold values indicate statistically significant results (*p*-value < 0.05)

*mDISCERN* modified DISCERN

There was no statistically significant difference between the group with an mDISCERN score ≥ 3 and the group with an mDISCERN score < 3 concerning video upload duration on YouTube™, number of views, comments, likes, and subscribers. The videos with higher mDISCERN scores were significantly more likely to be produced by doctors (66% vs 33%) when compared to those with lower mDISCERN scores.

There was not a significant difference between the two groups regarding the video being suggested to patients (*p*-value = 0.23). However, a higher percentage of videos with mDISCERN ≥ 3 (21%) was suggested for resident doctors compared to those with mDISCERN < 3 (2%). This difference was statistically significant (*p*-value = 0.006).

Notably, none of the videos with a higher mDISCERN score were produced by patients, while 11% of the videos with a lower score were patient-produced.

Table 6 showcases the general features of videos based on their Global Quality Scale (GQS) scores, divided into two categories: score < 3 and score ≥ 3. It appears that the median upload duration on YouTube™ was longer for videos with a GQS score below 3. In contrast, the median video length was longer for those scoring three or higher. However, the two groups had no statistically significant differences regarding these aspects and the number of views, comments, likes, and subscribers, as suggested by the provided *p*-values.

**Table 6:** Comparison of video features according to GQS score

	GQS < 3 (*N* = 59)	GQS ≥ 3 (*N* = 31)	*P*-value
Duration of upload on YouTube (days)			
Median [Q1, Q3]	1880 [942, 3330]	1190 [685, 1890]	0.14
Video length (sec)			
Median [Q1, Q3]	261 [116, 638]	441 [279, 1700]	**0.03**
Nr. views			
Median [Q1, Q3]	16,300 [1750, 60,200]	14,700 [1160, 36,400]	0.44
Nr. comments			
Median [Q1, Q3]	6.00 [1.00, 19.3]	12.0 [1.00, 33.0]	0.74
Nr. likes			
Median [Q1, Q3]	66.0 [13.5, 242]	156 [7.50, 402]	0.28
Nr. Subscribers			
Median [Q1, Q3]	9850 [778, 78,400]	13,100 [1240, 58,300]	0.53
Production			0.25
Doctors	24 (41%)	15 (48%)	
Professional channels	17 (29%)	9 (29%)	
Hospitals	11 (19%)	7 (23%)	
Patients	7 (12%)	0 (0%)	
Suggested to patients?			** < 0.001**
No	59 (100%)	22 (71%)	
Yes	0 (0%)	9 (29%)	
Suggested to young trainees/resident doctors?			**0.01**
No	58 (98%)	25 (81%)	
Yes	1 (2%)	6 (19%)	

Bold values indicate statistically significant results (*p*-value < 0.05)

*GSQ* Global Quality Scale

When considering the production source, there was a larger percentage of doctor-produced videos in the group with GQS scores of 3 or higher (48%), although this difference was not statistically significant (*p*-value = 0.25). Notably, patient-produced videos only appeared in the group with GQS scores of less than three.

Importantly, all videos with a GQS score less than three were not recommended to patients, while 29% of videos with a GQS score of 3 or more were, representing a significant difference (*p*-value < 0.001). A similar pattern was observed for recommendations to resident doctors, with 2% of the videos with a GQS score of less than 3 and 19% with a GQS score of 3 or more recommended. This difference was also statistically significant (*p*-value = 0.01).

## Discussion

This study is the first to focus on YouTube™ videos about hysteroscopy. We scrutinized 90 videos, each averaging nearly 96,000 views. Considering the increasing reliance of patients on online resources for health information, it’s plausible that a significant portion of these views came from individuals seeking insight into hysteroscopy procedures [1–7]. Despite 83 of these 90 videos being produced by medical experts (hospitals, doctors, or professional medical channels), our evaluation found them lacking in terms of scientific reliability, clarity, and comprehensiveness, a significant revelation considering the growing role of YouTube™ in health information dissemination [3, 5, 7].

This finding aligns with similar analyses conducted on YouTube™ videos regarding other surgical procedures, such as robotic myomectomy [30], uterine leiomyoma surgeries [31] and hysterectomy [32]. These investigations, involving the assessment of 150, 137, and 66 videos respectively, underscored that YouTube™ might not be an optimal platform for disseminating accurate and comprehensible medical information to the public.

Although the focus of this study was restricted to YouTube™, a broader examination of other online platforms like Google™, Facebook™, LinkedIn, Instagram, and YouTube™ demonstrated a similar tendency [33]. A study exploring treatment options for overactive bladder syndrome revealed a discernible information gap across these platforms, with search results predominantly occupied by homeopathic and alternative medicine. This scenario underscores the need for trustworthy digital health information [33].

Furthermore, our analysis found that only a minimal fraction of the videos (7 out of 90) offered value to trainees, echoing an earlier study on obstetric and gynecological physical examinations, which found only 29 out of 176 videos analyzed useful for self-guided learning among medical students [34]. This implies that YouTube™ might not be an optimal learning tool for medical students and early-career practitioners, particularly in gynecology.

A troubling observation from our study was that none of the scrutinized videos achieved the minimum score on mDISCERN and GQS scales. This lack of quality was consistent regardless of whether the videos were produced by experts or not, underscoring that being an expert does not guarantee the delivery of reliable, clear, and comprehensive information. This could lead to misinformation or misunderstanding among patients seeking information about hysteroscopy online, subsequently affecting their decision-making process and causing unwarranted anxiety.

Preoperative anxiety, linked to a lack of information or misinformation, can influence patients’ emotional states, potentially exacerbating pain perception during the procedure [35–37]. Despite the minimally invasive nature of hysteroscopy, it’s essential to acknowledge that anxiety is not always avoidable and could be notably intense in women [38]. This anxiety, occasionally manifesting as increased pain sensitivity, can be triggered by various factors, including cervical dilatation, intrauterine pressure, manipulation, and emotional states [38–40]. Technological advancements have ushered in an era of new and smaller surgical devices, significantly contributing to the field of hysteroscopy. In particular, miniaturized mechanical instruments have been developed to optimize precision and efficacy across hysteroscopic procedures [41]. These technological advancements, alongside the diffusion of the vaginoscopic ‘no touch’ technique that obviates the need for cervical manipulation with a speculum and tenaculum, alleviate potential pain and discomfort [42]. Pharmacological and non-pharmacological measures can be strategically employed in selected cases to manage pain and facilitate the examination process [43–46]. Pharmacological interventions such as local anesthetics, non-steroidal anti-inflammatory drugs (NSAIDs), and cyclooxygenase-2 inhibitors have been found to be efficacious when discomfort arises [47]. These strategies include warming the distension medium, music diffusion, continuous procedural updates, or enabling the patient to view the procedure on a monitor [43–47]. Furthermore, enhancing doctor-patient communication and comprehensive patient education are practical non-pharmacological interventions to reduce preoperative anxiety and improve patient satisfaction [48, 49]. However, the efficacy of these strategies could be undermined if the information available online, as our study indicates, is either insufficient or misleading. Therefore, providing accurate information from experts becomes paramount to prevent patients from seeking potentially incorrect information online and ensure a successful hysteroscopy procedure.

## Conclusions

While our study provides valuable insights, we must acknowledge its limitations. It did not evaluate all available videos about hysteroscopy, and YouTube™’s dynamic nature means the pool of videos may have changed since our research was conducted. We only considered videos in English, potentially limiting the generalizability of our findings. Moreover, we did not explore the effect of video content on patient outcomes or satisfaction, which should be the focus of future research.

Despite these limitations, the study underscores the need for accurate, reliable, high-quality online medical information. Relying solely on YouTube™ for patient education can potentially lead to misconceptions and unnecessary anxiety. As such, hysteroscopy experts need to harness digital platforms’ power and deliver reliable, high-quality information to improve patient understanding and overall procedure success.

Guiding patients to selected, expert-approved videos can enhance their understanding, alleviate anxiety, and increase the success rate of procedures. Conversely, leaving patients uninformed or allowing them to find potentially misleading information can lead to stress and potential procedure failure.

## Data Availability

The data that support the findings of this study are available on request from the corresponding author [SGV].
